# Seasonal Succession of Bacterial Communities in Three Eutrophic Freshwater Lakes

**DOI:** 10.3390/ijerph18136950

**Published:** 2021-06-29

**Authors:** Bin Ji, Cheng Liu, Jiechao Liang, Jian Wang

**Affiliations:** Department of Water and Wastewater Engineering, Wuhan University of Science and Technology, Wuhan 430065, China; binji@wust.edu.cn (B.J.); liucheng0329@126.com (C.L.); jiechaoliang2021@126.com (J.L.)

**Keywords:** eutrophication, bacterial community, 16S rRNA gene, seasonal changes, bacterial eutrophic index, nitrogen to phosphorus ratio

## Abstract

Urban freshwater lakes play an indispensable role in maintaining the urban environment and are suffering great threats of eutrophication. Until now, little has been known about the seasonal bacterial communities of the surface water of adjacent freshwater urban lakes. This study reported the bacterial communities of three adjacent freshwater lakes (i.e., Tangxun Lake, Yezhi Lake and Nan Lake) during the alternation of seasons. Nan Lake had the best water quality among the three lakes as reflected by the bacterial eutrophic index (BEI), bacterial indicator (*Luteolibacter*) and functional prediction analysis. It was found that *Alphaproteobacteria* had the lowest abundance in summer and the highest abundance in winter. *Bacteroidetes* had the lowest abundance in winter, while *Planctomycetes* had the highest abundance in summer. N/P ratio appeared to have some relationships with eutrophication. Tangxun Lake and Nan Lake with higher average N/P ratios (e.g., N/P = 20) tended to have a higher BEI in summer at a water temperature of 27 °C, while Yezhi Lake with a relatively lower average N/P ratio (e.g., N/P = 14) tended to have a higher BEI in spring and autumn at a water temperature of 9–20 °C. BEI and water temperature were identified as the key parameters in determining the bacterial communities of lake water. Phosphorus seemed to have slightly more impact on the bacterial communities than nitrogen. It is expected that this study will help to gain more knowledge on urban lake eutrophication.

## 1. Introduction

Urban lakes play an irreplaceable role in regional environments for humanity and nature [1]. Urban lakes can maintain biodiversity, adjust the temperature, balance the water in the air and soil, increasing air humidity and alleviating storm flooding [2]. In addition, urban lakes can be simply deemed a kind of scenery of the city. However, with the human activity of urban development, the detrimental effect on urban lake environment has become increasingly prominent [3]. The deterioration of the lake environment is usually associated with the eutrophication [4], which is mainly caused by the excessive input of nitrogen and phosphorus [5] from the discharge of domestic wastewater in cities [6]. Hence, knowing the water quality of urban lakes is favorable to prevent eutrophication.

It is well known that microorganisms play an important role in material and energy conversion and metabolism in aquatic ecosystems [7,8]. Bacteria are widespread in aquatic ecosystems and can contribute to the self-purification of water bodies. The shifts in the planktonic bacterial colony can somewhat reflect the water environment [9,10]. Thus, the bacterial community plays a fundamental role in environmental processes and geochemical cycles [11]. Although pure culture methods have been applied traditionally for bacteria identification [12], many microorganisms are viable but non-culturable [13]. In recent years, with the progress of sequencing technology, more and more studies on the microbial communities of environmental samples have been based on a molecular level sequencing [14,15]. Illumina MiSeq sequencing technology has gained attention due to its accuracy [16], universality and effectiveness, and has been widely applied in the identification of microbial communities of varied environmental samples [17,18,19].

For urban lakes, sediment samples are usually used to reveal the microbial component [20]. In fact, microbes in the lake water can reflect the water quality directly. With the change of seasons, the water level and the microbial community structure would change [21,22], while the temperature can also impact on the microbial community in an ecosystem [23]. However, few studies have reported on the aquatic bacterial compositions in adjacent urban lakes in different seasons, which seems to make the knowledge on urban lake eutrophication incomplete. Therefore, it is necessary to examine the microbial community compositions of adjacent urban lakes in different seasons.

Wuhan, located in the middle reaches of the Yangtze River and the east of Jianghan Plain, has experienced rapid urbanization with drastic deterioration of lake environments [24]. This study reported the bacterial communities of surface water in three adjacent lakes in Wuhan, which are distributed in the Hongshan district and composed with mainly leached soil with subtropical evergreen broad-leaved forest vegetation. The seasonal water qualities and successions of bacterial community structure were systematically analyzed. This study is expected to gain knowledge on urban lake eutrophication in different seasons.

## 2. Materials and Methods

### 2.1. Study Area and Sample Collection

**Figure 1:**
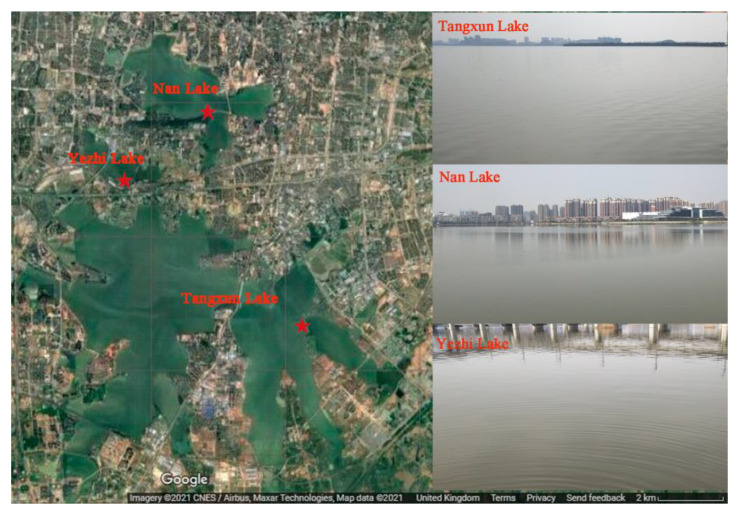
A map with the location of the three lakes and sampling sites.

The water samples were collected from three adjacent freshwater lakes (i.e., Tangxun Lake, Yezhi Lake and Nan Lake) in Wuhan, China. Tangxun Lake is the largest urban lake in Asia, with an area of 52.2 km^2^, while Nan Lake and Yezhi Lake are much smaller, with water areas of 7.6 and 1.6 km^2^, respectively. The surface water samples (top 30 cm) were collected on sunny days on 20 June 2018 (summer), 26 September 2018 (autumn), 27 December 2018 (winter) and 8 March 2019 (spring). The water temperatures were about 9, 27, 20 and 3 °C for spring, summer, autumn and winter, respectively. Representative locations along with the scenery of the three stations for surface water sampling are depicted in Figure 1. The water samples in spring, summer, autumn, and winter are denoted as JCTXL, JXTXL, JQTXL and JDTXL for Tangxun Lake; JCYZL, JXYZL, JQYZL and JDYZL for Yezhi Lake and JCNL, JXNL, JQNL and JDNL for Nan Lake. One liter of water was collected via a water sample collector. The collected water samples were stored temporarily in plastic bottles in a foam box packed with ice bags. The collected water samples were analyzed within 4 h.

### 2.2. Determination of Water Quality Indices

The chemical oxygen demand (COD), total nitrogen (TN) and total phosphorus (TP) were determined in accordance with the standard method [25] in triplicate. The equipment applied in this study included a YXQ-LS-30 SII sterilizer (Boxun Instrument, Shanghai, China), an A580 spectrophotometer (AOE Instruments A580, Shanghai, China) and a SC-2 COD detector (Huaxia, Beijing, China). A pH meter (OHAUS, ST3100F) was used to measure pH and temperature.

The trophic state index (TSI) was determined as follows [26,27]:

TSI(TN) = 54.45 + 14.43 ln(TN)

TSI(TP) = 14.42 ln(TP) + 4.15

$$\mathrm{TSI}=\frac{\mathrm{TSI}\left(\mathrm{TN}\right)+\mathrm{TSI}\left(\mathrm{TP}\right)}{2}$$

where TN and TP were in mg·L^−1^ and mg·m^−3^, respectively.

Bacterial eutrophic index (BEI) can be determined as follows [28]:

$$\mathrm{BEI}=\frac{\mathrm{Cyano}-A}{\mathrm{Actino}-A}$$

where Cyano-A and Actino-A were the relative abundance of *Cyanobacteria* and *Actinobacteria*, respectively. The respective relative abundances of *Cyanobacteria* and *Actinobacteria* were derived from Miseq sequencing.

The modified BEI (BEI′) considered the impact of temperature (T), which can be calculated as follows [29]:

$$\mathrm{BEI}\prime =\frac{\mathrm{Cyano}-A}{\mathrm{Actino}-A}\left(\frac{1}{-0.1065+0.09732\;T-0.002530{\;T}^{2}}\right)$$

### 2.3. Microbial Community Analysis

100 mL water samples were filtered through a 25 mm diameter, 0.2 μm filter (Osmonics, Livermore, CA, USA), and the filters were kept at −20 °C. Total DNA was extracted with the E.Z.N.A. soil DNA separation kit (OMEGA Biotek Inc., Norcross, GA, USA) following the manufacturer’s instructions.

The V4 region of bacterial 16S rRNA genes was amplified using a primer set of 520F-802R [30]. The Illumina Miseq platform was applied for the paired-end sequencing of the DNA fragments. The sequence analyses were conducted as described [31]. The 16S rRNA gene sequence can be obtained from the NCBI website (http://www.ncbi.nlm.nih.gov/sra/, PRJNA631438, accessed on 22 June 2020).

## 3. Results and Discussion

### 3.1. Water Properties

Table 1 depicts the water quality of the three lakes in four seasons, indicating that the water quality indicators of the three freshwater lakes were different. Obviously, all of the three lakes were suffering serious pollution, especially from nitrogen and phosphorus. According to the “Environmental Quality Standard for Surface Water” (GB3838-2002), the Class V lakes should have nitrogen and phosphorus contents of no more than 2.0 and 0.2 mg/L, respectively [32]. The water quality of the three lakes was inferior to the Class V lakes. Among them, the water quality of Tangxun Lake was worst due to its highest average values of TN (7.59 mg/L) and TP (0.36 mg/L). The average TSI values for Tangxun Lake, Nan Lake and Yezhi Lake were calculated to be 86, 77 and 74, respectively, which further indicated that Tangxun Lake had the highest nitrogen and phosphorus contents. However, the Nan Lake had a highest average COD value with 31.69 mg/L. The average N/P ratios for Tangxun Lake, Yezhi Lake and Nan Lake were 20.80, 13.57 and 20.24, respectively, suggesting that Yezhi Lake was rather different from Tangxun Lake and Nan Lake with a relatively lower N/P ratio.

The water indicators of the same lake in different seasons were also different. It indicated that the nitrogen and phosphorus concentrations were relatively higher in lake waters in summer compared to other seasons. Although there is usually plenty of rain in summer in Wuhan, which can dilute the lake water and lower the nutrients levels, the rain can also bring pollutants from air and land to the lake water. In addition, the high temperatures could also contribute to the release of nutrients from sediments [33], leading to the increase in nutrient concentrations in the water.

### 3.2. Operational Taxonomic Units, Bacterial Abundance and Diversity

**Figure 2:**
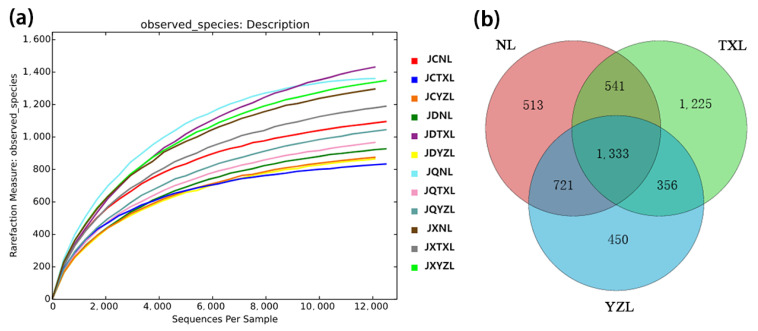
(**a**) Rarefaction curves of operational taxonomic units; (**b**) Venn diagram. The water samples in spring, summer, autumn, and winter, respectively, are denoted as JCTXL, JXTXL, JQTXL and JDTXL for Tangxun Lake, JCYZL, JXYZL, JQYZL and JDYZL for Yezhi Lake and JCNL, JXNL, JQNL and JDNL for Nan Lake.

Figure 2a indicates that the sequencing depth was sufficient to assess the bacterial community. Among the 12 samples, about half of the OTUs were not shared as shown in Figure 2b, indicating that the bacterial communities in the three freshwater lakes were different. On the contrary, the three lakes shared 1333 OTUs, possibly because they were adjacent.

Table 2 lists the alpha diversity indices at the same sequencing depth, including diversity indices (Shannon and Simpson) and richness indices (ACE and Chao1). It indicated that the richness of bacterial community of Tanxun Lake in winter was the highest. On the contrary, the diversity of bacterial community of Nan Lake in autumn was the highest.

### 3.3. Seasonal Microbial Community Structure

**Figure 3:**
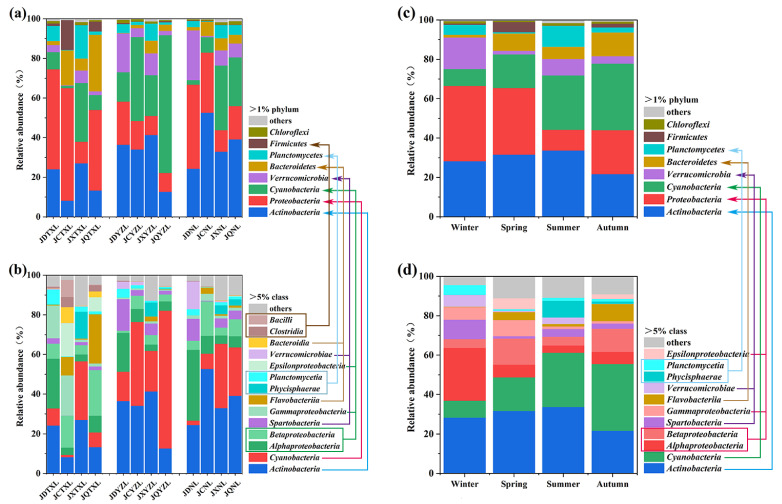
Bacterial community of each lake at phylum level (**a**) and class level (**b**); overall bacterial community of each season at phylum level (**c**) and class level (**d**). The water samples in spring, summer, autumn, and winter, respectively, are denoted as JCTXL, JXTXL, JQTXL and JDTXL for Tangxun Lake; JCYZL, JXYZL, JQYZL and JDYZL for Yezhi Lake and JCNL, JXNL, JQNL and JDNL for Nan Lake.

The three lakes have their specific microbial community compositions in different seasons as depicted clearly in Figure 3a,b. Taking the average levels of the four seasons, the main phyla were *Proteobacteria* (39.7%), *Actinobacteria* (18.2%), *Bacteroidetes* (13.6%) and *Cyanobacteria* (11.8%) for Tangxun Lake. For Yezhi Lake, the main components were *Cyanobacteria* (36.8%), *Actinobacteria* (31.1%), *Proteobacteria* (13.8%) and *Verrucomicrobia* (9.3%). For Nan Lake, the main components were *Actinobacteria* (37.3%), *Proteobacteria* (25.2%), *Cyanobacteria* (16.8%) and *Verrucomicrobia* (10.1%). The main phyla of the lake water samples were quite similar to previous reports on freshwater [21,34].

Some bacteria had a distinct altering rule for their abundance with regard to different seasons. For example, the phylum of *Proteobacteria*, usually had the lowest abundance in summer and the highest abundance in winter (Figure 3c), which was similar to a previous report on Lake Taihu [21]. At a finer scale, the abundance of *Alphaproteobacteria* varied positively with low temperatures as displayed in Figure 2b, implying the excellent adaptation of *Alphaproteobacteria* to low temperature. Indeed, the *Alphaproteobacteria* were previously found to be dominant in cold environments [35,36]. On the contrary, *Bacteroidetes* tended to have the lowest abundance in winter (3 °C), while the *Planctomycetes* tended to have the highest abundance in summer (27 °C). In fact, the anammox *Planctomycetes* could grow well at around 30 °C [37,38], while the lab-scale anammox process could be operated at 25 °C [39].

**Figure 4:**
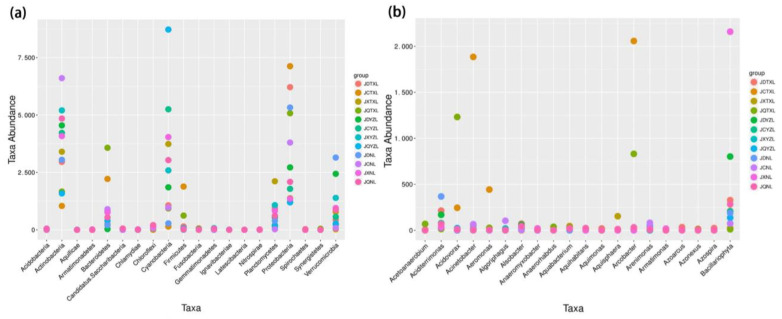
Obvious different abundance in bacterial taxa at phylum level (**a**) and genus level (**b**). The water samples in spring, summer, autumn, and winter, respectively, are denoted as JCTXL, JXTXL, JQTXL and JDTXL for Tangxun Lake; JCYZL, JXYZL, JQYZL and JDYZL for Yezhi Lake and JCNL, JXNL, JQNL and JDNL for Nan Lake.

Figure 4 depicts the OTUs of bacteria that differ significantly at the phylum and genus levels. The phyla with most obviously different abundances were found to be *Actinobacteria*, *Cyanobacteria* and *Proteobacteria* among all the water samples. Specifically, the greatest abundance of *Actinobacteria*, *Cyanobacteria* and *Proteobacteria* of water samples were found in Nan Lake in spring, Yezhi Lake in autumn and Tangxun Lake in spring, respectively. At the genus level, bacteria with the most obvious different abundance were found to be *Acinetobacter*, *Arcobacter* and *Bacillariophyta* among all the water samples.

### 3.4. Eutrophication Assessment from the Microbial Aspect

The average BEI and BEI’ indexes shown in Table 3 indicated that Yezhi Lake had the highest bacterial eutrophic index, which was different from the results from the TSI indexes, where Tangxun Lake had a highest TSI on average. This can be attributed to the fact that the N/P ratio of Yezhi Lake was obviously lower than that of Tangxun Lake and Nan Lake, suggesting that the Yezhi Lake had relatively higher phosphorus content. In fact, phosphorus was deemed the most vital element for the eutrophication of shallow lakes [40]. This gives a plausible explanation for the different results of the BEI and TSI assessment methods.

**Figure 5:**
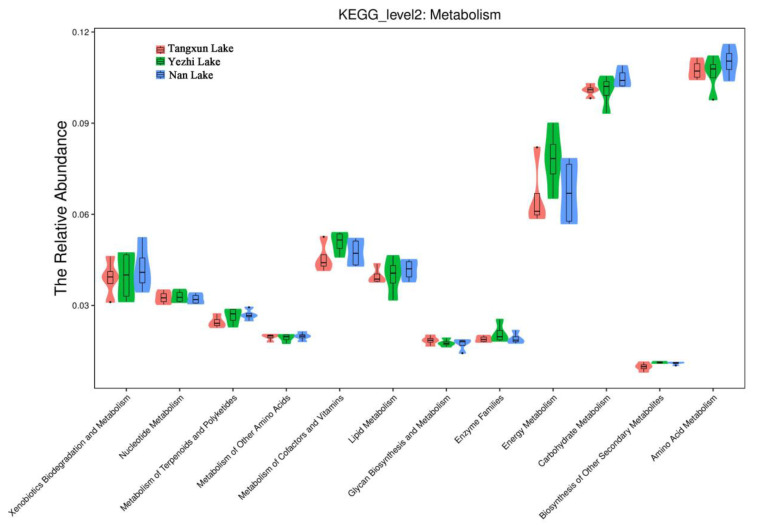
Function prediction of bacterial taxa based on the second level KEGG module by PICRUSt.

It has been reported that *Luteolibacter* in the surface water of fresh lakes in winter is a potential bacterial indicator of good-quality lakes [41]. In this study, the abundance of *Luteolibacter* of Tangxun Lake, Yezhi Lake and Nan Lake in winter was 0.19, 3.43 and 12.03%, respectively. This suggested that Nan Lake had the best water quality among the three eutrophic lakes. In fact, it can be seen from Figure 5 that the bacteria in Nan Lake had higher function than the other two lakes in terms of substrate metabolism, such as carbohydrate metabolism, amino acid metabolism, lipid metabolism, etc. This also indicated that Nan Lake had the strongest self-purification ability among the three lakes.

### 3.5. Relationship between N/P Ratio and Seasonal Eutrophication

Moreover, we tentatively put forward that lakes with a relatively higher N/P ratio (e.g., N/P = 20) tend to have a higher average BEI in summer at a water temperature of 27 °C, while lakes with a relatively lower N/P ratio (e.g., N/P = 14) tend to have a higher average BEI in spring and autumn at a water temperature of 9–20 °C. To avoid eutrophication, we should lower the N/P ratio of lakes during spring and autumn, while increasing the N/P of lakes during summer. This is in agreement with a previous study on controlling the cyanobacterial blooms in Taihu Lake [5,42], e.g., the sole N addition had a significant positive effect on phytoplankton growth in summer [5]. However, more evidence should be presented on this viewpoint from global lakes.

### 3.6. Relationships between Bacterial Community and Water Quality

**Figure 6:**
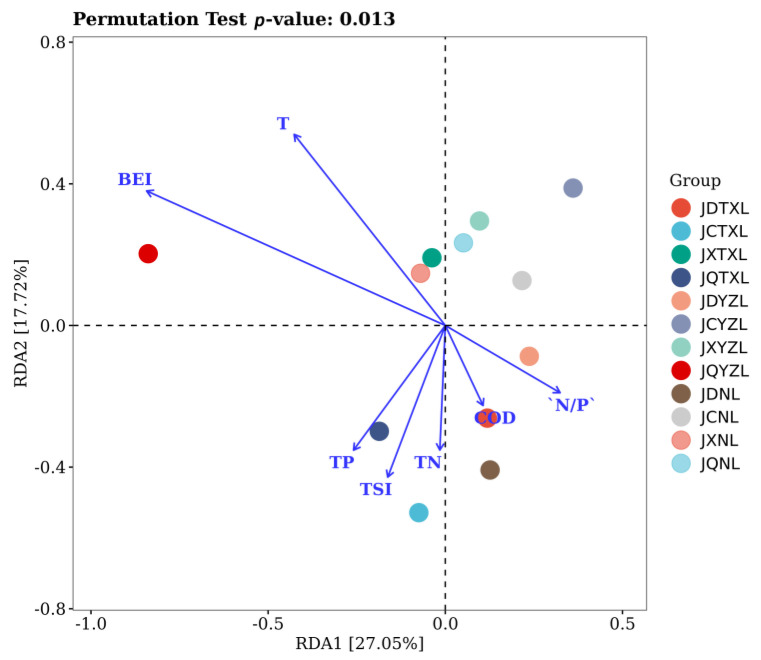
RDA analysis showing the relationships between bacterial communities and nutrient parameters. The water samples in spring, summer, autumn, and winter, respectively, are denoted as JCTXL, JXTXL, JQTXL and JDTXL for Tangxun Lake; JCYZL, JXYZL, JQYZL and JDYZL for Yezhi Lake and JCNL, JXNL, JQNL and JDNL for Nan Lake.

RDA analysis was applied to look into the relationship between bacterial community and water quality. As shown in Figure 6, BEI and temperature were the most significant parameters for shaping the bacterial communities of lake water, reflected by the length of the arrow-lines. Indeed, BEI can be a feasible bacterial index for eutrophic assessment of freshwater lakes [28], which is significantly impacted by temperature [29]. This can be ascribed to the abundance of aquatic bacteria (e.g., cyanobacteria) that can reflect the water quality of lakes, which is impacted by the temperature. Other research results also indicated that temperature was a key factor affecting the bacterial abundance in water [43]. Moreover, it appears that the BEI had a positive correlation with temperature, and that TN had a positive correlation with TP. Although both phosphorus and nitrogen have been deemed as the most important parameters for eutrophication, TP seemed to have a slightly greater impact on shaping the bacterial community in lake water than TN in this study.

## 4. Conclusions

This study reported the seasonal bacterial compositions of surface water of three adjacent lakes in Wuhan, i.e., Tangxun Lake, Yezhi Lake and Nan Lake. It was found that Nan Lake had the best water quality among the three lakes as reflected by the BEI, bacterial indicator of *Luteolibacter* and functional prediction. Bacterial alternation rules were disclosed. *Alphaproteobacteria* had the lowest abundance in summer and the highest abundance in winter. *Bacteroidetes* tended to have the lowest abundance in winter, while *Planctomycetes* tended to have the highest abundance in summer. Moreover, the N/P ratio appeared to have a relationship with seasonal eutrophication of freshwater lakes. A high N/P ratio favored eutrophication in summer while a low N/P ratio favored eutrophication in spring and autumn evidenced by the BEI values.

## Figures and Tables

**Table 1:** Water quality indicators of the three lakes in each season.

	Sample ID	Season	COD (mg/L)	TN (mg/L)	TP (mg/L)
Tangxun Lake	JDTXL	Winter	24.28	9.16	0.35
	JCTXL	Spring	26.28	6.22	0.35
	JXTXL	Summer	34.32	9.42	0.42
	JQTXL	Autumn	25.11	5.56	0.33
Yezhi Lake	JDYZL	Winter	12.14	2.38	0.21
	JCYZL	Spring	15.62	2.54	0.12
	JXYZL	Summer	15.16	3.01	0.28
	JQYZL	Autumn	14.22	2.43	0.22
Nan Lake	JDNL	Winter	32.38	4.18	0.18
	JCNL	Spring	30.39	2.29	0.16
	JXNL	Summer	33.45	5.16	0.28
	JQNL	Autumn	30.54	4.75	0.19

**Table 2:** The seasonal α-diversity indices of the bacterial community of the three lakes.

	Sample ID	Chao1	ACE	Simpson	Shannon
Tangxun Lake	JDTXL	1622.14	1722.66	0.970521	7.85
	JCTXL	935.81	915.27	0.982499	7.65
	JXTXL	1309.40	1375.19	0.984909	7.97
	JQTXL	1203.57	1152.77	0.934474	6.80
Yezhi Lake	JDYZL	940.40	985.55	0.968654	7.04
	JCYZL	944.04	977.53	0.967030	7.02
	JXYZL	1469.94	1530.30	0.990195	8.44
	JQYZL	1184.99	1239.24	0.978945	7.52
Nan Lake	JDNL	990.65	1037.55	0.954592	6.89
	JCNL	1232.44	1238.85	0.989097	8.12
	JXNL	1470.83	1492.79	0.985983	8.30
	JQNL	1363.01	1374.30	0.993332	8.78

**Table 3:** Eutrophic index of the three lakes in different seasons.

Index	Season	Tangxun Lake	Yezhi Lake	Nan Lake
N/P	Spring	17.77	21.17	14.31
	Summer	22.43	10.75	18.43
	Autumn	16.85	11.05	25.00
	Winter	26.17	11.33	23.22
	AVG	20.80	13.57	20.24
TSI	Spring	84.72	70.54	71.87
	Summer	89.03	77.88	81.77
	Autumn	83.49	74.59	78.37
	Winter	87.52	74.11	77.06
	AVG	86.19	74.28	77.27
BEI	Spring	0.14	1.24	0.15
	Summer	1.10	0.50	0.99
	Autumn	0.56	5.49	0.63
	Winter	0.36	0.41	0.09
	AVG	0.54	1.91	0.46
BEI’	Spring	0.25	2.20	0.26
	Summer	1.62	0.74	1.46
	Autumn	0.68	6.64	0.76
	Winter	2.22	2.50	0.57
	AVG	1.19	3.02	0.76
Abundance of *Luteolibacter* (%)	Winter	0.19	3.43	12.03

## Data Availability

Data available in a publicly accessible repository.
